# Face memory and face perception in autism

**DOI:** 10.1177/13623613211027685

**Published:** 2021-06-23

**Authors:** Mirta Stantić, Eri Ichijo, Caroline Catmur, Geoffrey Bird

**Affiliations:** 1 University of Oxford, UK; 2 King’s College London, UK

**Keywords:** CFMT, face memory, face perception, GFMT, OFMT

## Abstract

It has been argued that autistic individuals have difficulties with face memory but typical face perception. However, only one previous study has examined both face memory and face perception in the same individuals, and this study was conducted with a small group of autistic children. Here, face recognition was examined with a group of autistic adults using two face perception tasks (including one designed to avoid a neurotypical bias) and a standard test of face memory. Self-reported face recognition difficulties in everyday life were also recorded. The group of adults with autism scored lower than a matched neurotypical control group on all face tasks and reported more problems with face recognition in everyday life. On the whole, results suggest difficulties with both face perception and face memory in autistic adults, although it should be noted that a wide range of scores were recorded from the autistic individuals, with some scoring in the neurotypical range.

**Lay abstract:**

It is well known that some people with autism have difficulties recognizing faces. It is generally thought that this is not because autistic individuals cannot perceive faces, but because autistic individuals have greater problems than people without autism in remembering faces. Here, we worked with a group of autistic adults and a group of non-autistic adults to test their ability to perceive and remember faces. We also asked each person to report any difficulties that they have with recognizing faces in everyday life. We find that, as a group, people with autism have difficulties with both remembering and perceiving faces, and report more problems recognizing faces in everyday life. However, it is worth noting that we observed a wide range of scores in the group of people with autism, with some autistic participants scoring as well as the group of people without autism.

The recognition of an individual via their face is a fundamental aspect of everyday social interaction. Face recognition has therefore been relatively well studied in individuals diagnosed with autism spectrum disorder (henceforth ‘autism’), a condition defined by persistent challenges; difficulties or alterations in social communication and social interaction; and restricted, repetitive patterns of behaviour, interests or activities (American Psychiatric Association, 2013). Across approximately 150 studies, results are mixed, with reports of both typical and impaired face recognition in autism. In their review, Weigelt et al. (2012) argue that these mixed results can largely be explained by a selective deficit in face memory in the presence of intact face perception. With some exceptions (see Tang et al., 2015), studies are in accordance with this view – the majority of studies requiring a face to be held in memory and to be compared to a subsequent exemplar reveal performance impairments in autistic volunteers, whereas studies involving tasks requiring two faces to be compared without memory demands (e.g. in a simultaneous identity-matching task) tend to find typical performance by autistic volunteers. Problematically, however, as far as we are aware, there is only one previous study that compares face memory and face perception in the same autistic individuals (Gepner et al., 1996), and this study tested the performance of only seven children with autism. It is important to test face perception and face memory in the same individuals because differential performance across groups of individuals in different studies may be explained by sample differences in, for example, age or gender ratio or differences in experimental stimuli, rather than the type of face processing required for good performance.

This study therefore examined the performance of a group of 31 autistic adults on a standard test of face memory (the Cambridge Face Memory Test (CFMT); Duchaine & Nakayama, 2006) and a standard test of face perception (the Glasgow Face Matching Test (GFMT); Burton et al., 2010). In addition, a novel test of face perception was used, the Oxford Face Matching Test (OFMT; Stantić et al., 2021), which was specifically designed to provide a non-biased test of face perception for atypical groups. The OFMT presents face matching trials (participants are required to determine whether two face images are of the same person) where the difficulty is determined using facial recognition algorithms. The use of algorithms allows the full range of difficulty to be sampled in a way that does not favour the processing strategies of any one group (e.g. neurotypical (NT) or autistic). For example, if autistic individuals are less likely to process faces holistically than NT individuals (Joseph & Tanaka, 2003), and NT performance is used to calibrate the difficulty of stimulus items such that on average NT individuals get 75% correct, the set of particular stimuli selected may produce better or worse performance in a group of autistic individuals (if those stimuli are easier/harder to distinguish based on local features) even if the autistic individuals are as good as NT individuals on an infinitely large set of face stimuli.

## Methods

### Participants

Thirty-one autistic individuals (9 females, mean age = 34.4 years, *SD* age = 8.6 years, mean IQ = 110.6, *SD* IQ = 26.0) and 30 NT individuals (12 females, mean age = 34.5 years, *SD* age = 6.8 years, mean IQ = 111.1, *SD* IQ = 9.74) participated. There were no significant differences between the autism and NT groups in IQ (*t*(59) = 0.10, *p* = 0.92), age (*t*(59) = −0.09, *p* = 0.93) or gender (χ^2^ = 0.81, *p* = −0.37). Autistic individuals were diagnosed by an independent clinician and met criteria for autism or autism spectrum on the Autism Diagnostic Observation Schedule–Second Edition (ADOS-2; Lord et al., 2000). Autistic individuals reported the following co-occurring conditions within the last 5 years: anxiety (4 participants), depression (4), attention deficit hyperactivity disorder (4), dyspraxia (3) and obsessive-compulsive disorder (1). Three originally recruited NT participants were replaced as they (1) had a current or past psychiatric neurodevelopmental diagnosis, (2) used psychotropic meditation or (3) failed to attend to the task. All NT participants scored below cut-off (32) on the 50-Item Autism Spectrum Quotient (AQ-50; Baron-Cohen et al., 2021).

### Procedure

**Figure 1.**
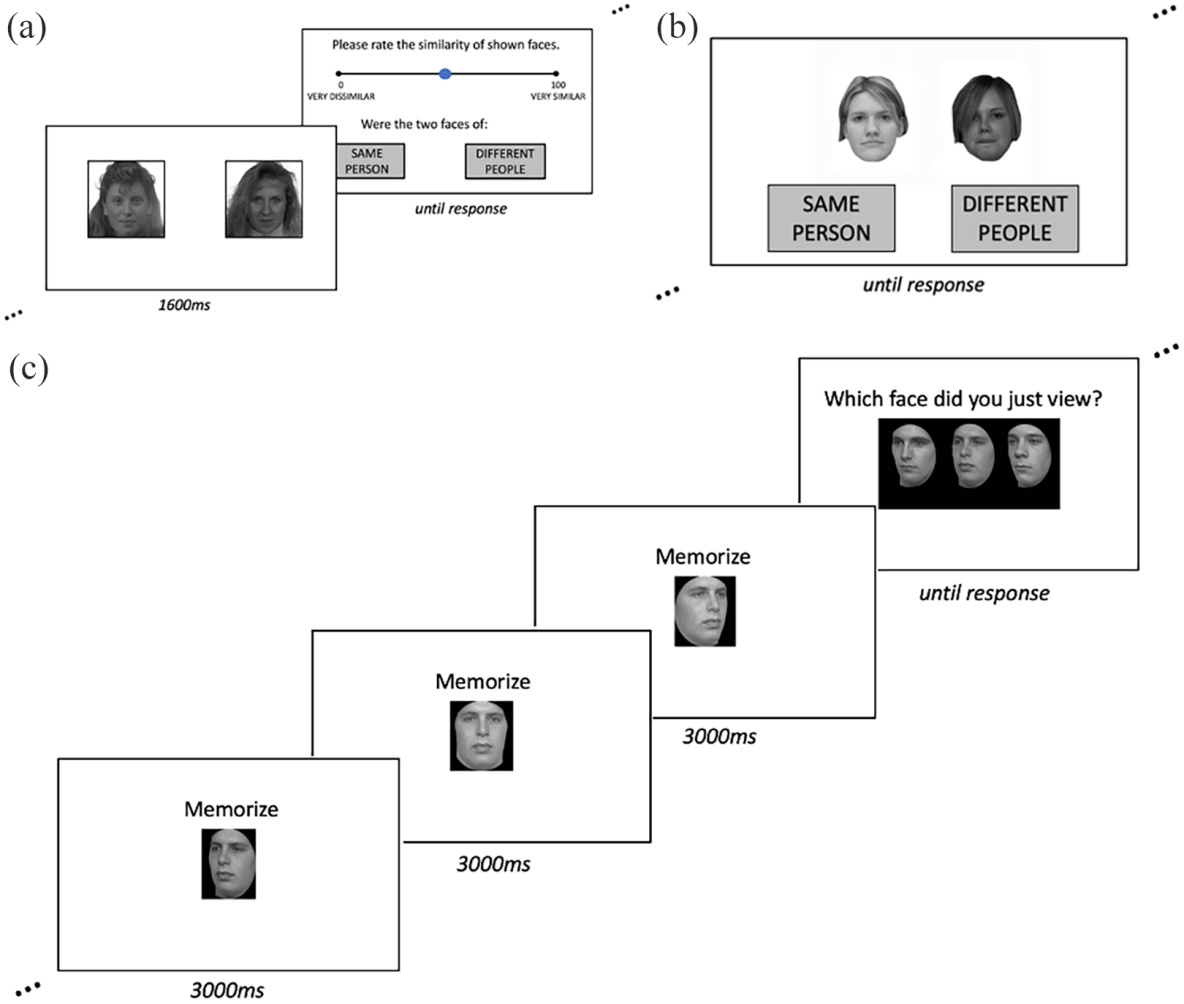
Illustration of sample trials for all three face processing tasks: (a) OFMT, a face matching task that presents faces for 1600 ms before participants have to rate the similarity of two faces and decide whether they were of the same person or different people; (b) GFMT, a face matching task that presents faces for an unlimited viewing time while participants decide whether the faces are of the same person or different people; and (c) CFMT, a face memory task during which participants learn faces from three viewpoints and subsequently select them from test displays with two foils (targets can be presented in identical or previously unseen variants, as well as with visual noise overlaid for difficulty).

Participants completed the CFMT, GFMT, OFMT and 20-Item Prosopagnosia Index (PI-20; Gray et al., 2017) in a randomized order. The CFMT is a test of face memory in which participants are initially required to learn six target faces. Across 72 trials, participants have to identify the learned identity among two distractors. The CFMT was developed as a diagnostic tool for developmental prosopagnosia (DP) and has good reliability (*r* = 0.67–0.70; Murray & Bate, 2020; Stantić et al., 2021; Wilmer et al., 2010) and excellent validity, as shown by its ability to discriminate between prosopagnosic and typical individuals. The GFMT (40 trials) and OFMT (200 trials) are tests of face perception using a matching task in which participants have to indicate whether two simultaneously presented face images are of the same individual or different individuals (see Figure 1 for task illustrations). Both tasks are reliable (GFMT *r* = 0.77; OFMT *r* = 0.75), and both show good validity in distinguishing between prosopagnosic and typical individuals, with the OFMT also able to distinguish between super-face-recognisers and typical individuals (Stantić et al., 2021). The OFMT was developed using facial recognition algorithms to determine item difficulty (instead of NT norming) to avoid a bias towards sensitivity in the NT population at the expense of atypical groups. PI-20 is a self-report questionnaire used to identify difficulties in face recognition in which increasing difficulty with face recognition is indicated by increasing scores. The study was approved by the local research ethics committee, and all authors report no conflicts of interest.

### Community involvement

Autistic people, both those who participated in this study and other volunteers in our laboratory, were asked to provide feedback on early versions of this study. The design of the study was adjusted based on the feedback provided. Upon publication, this research will be shared with people with autism who have expressed interest in being informed of the outcomes of this study.

## Results

**Figure 2.**
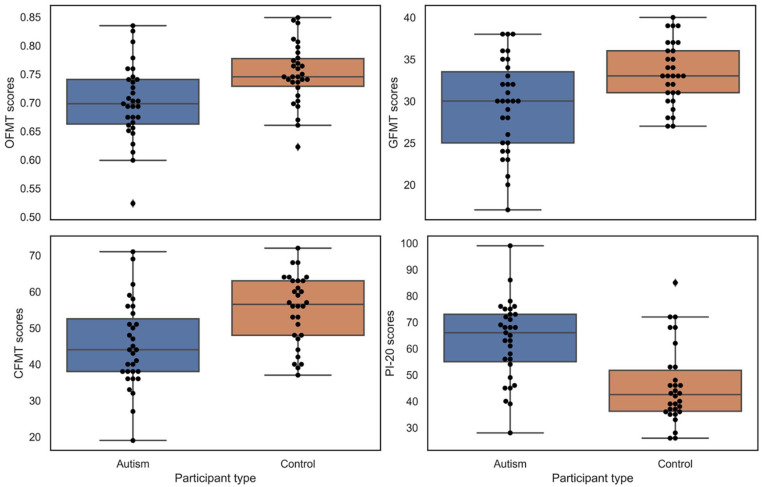
The difference between autistic (autism) and matched neurotypical (control) participants on all four tasks. The boxes represent interquartile scores, the horizontal lines in boxes represent group medians and the whisker lines span the full range of scores within each group (excluding any outliers, which are shown as separate dots). Matching tasks are shown in the top panel (OFMT, left; GFMT, right), whereas the bottom panel includes the memory task (CFMT, left) and the self-report measure of difficulties with face recognition (PI-20, right).

The results for both groups on all tests are shown in Figure 2. Relationships between all measures are included in Table 1.

**Table 1.** Relationships between all tasks separated by group (autism or control).

		CFMT	PI-20	GFMT	OFMT
CFMT	Autism	–			
	Control	–			
PI-20	Autism	–0.17	–		
	Control	–0.22	–		
GFMT	Autism	0.56**	0.09	–	
	Control	0.46**	–0.32	–	
OFMT	Autism	0.50**	0.20	0.70**	–
	Control	0.44*	–0.21	0.69**	–

CFMT: Cambridge Face Memory Test; PI-20: 20-Item Prosopagnosia Index; GFMT: Glasgow Face Matching Test; OFMT: Oxford Face Matching Test.

* Significance at the 0.05 level; **significance at the 0.01 level.

### CFMT

The performance of the autism group (*M* = 45.2, *SD* = 11.8, range = 19–71) was significantly worse than that of the NT group (*M* = 55.1, *SD* = 9.6, range = 37–72; *t*(59) =−3.60, *p* = 0.001). Of the 31 autistic participants, 26 (84%) scored below the median NT performance.

### GFMT

The performance of the autism group (*M* = 29.5, *SD* = 5.6, range = 17–38) was significantly worse than that of the NT group (*M* = 33.3, *SD* = 3.7, range = 27–40; *t*(59) =−3.15, *p* = 0.003). Of the 31 autistic participants, 22 (71%) scored below the median NT performance.

### OFMT

The performance of the autism group (*M* = 70.1%, *SD* = 6.6%, range = 52.4%–83.5%) was significantly worse than that of the NT group (*M* = 74.7%, *SD* = 5.6%, range = 62.3%–84.9%; *t*(59) =−2.94, *p* = 0.005). Of the 31 autistic participants, 24 (74%) scored below the median NT performance.

### PI-20

The autism group (*M* = 63.3, *SD* = 15.1) reported significantly more difficulties with face recognition than the NT group (*M* = 45.9, *SD* = 14.8; *t*(59) = 4.55, *p* < 0.00). Of the 31 autistic participants, 28 (90%) scored above the median NT score.

## Discussion

It has been claimed that autistic individuals are impaired on face memory but not face perception tasks (Weigelt et al., 2012, but see Tang et al., 2015). To test this claim, it is essential to test face memory and face perception in the same individuals, as the high heterogeneity seen in autism may mean that sampling differences (random or otherwise) across studies manifest as artefactual differences in performance across test types. Accordingly, a group of autistic adults completed a test of face memory (CFMT) and two tests of face perception (GMFT and OFMT). On each test, the group of autistic individuals scored lower than the NT control group and also reported more problems with face recognition on the PI-20.

These data are not consistent with claims that face perception is spared in autism, as performance on both the GFMT and the OFMT (with the latter designed to avoid a potential bias in favour of NT individuals) was lower in the autism group than the NT group. It is worth noting that impaired performance was not universal in the autism group, however, with nine and seven autistic individuals performing better than the median performance of the NT group on the GFMT and OFMT, respectively. Importantly, intact performance in some autistic individuals was not limited to face perception, but was also seen on the test of face memory where five autistic individuals performed better than the NT median. Interestingly, a single case of a participant with autism scoring below the NT median on the face perception tasks and above the NT median on the memory task (CFMT) was observed. This result highlights that the current data do not allow the source of any atypical performance in the autism group to be identified. It is unclear whether atypically good or poor performance is caused by atypical perception, attention or (general) memory. This is an important issue to be addressed by future research. Also, given that specific data on socioeconomic status and educational attainment were not recorded, we cannot be sure that this pattern of results would hold across any group of autistic individuals. Furthermore, the small sample of women in our population does not provide sufficient power to determine whether any of these effects interact with gender.

It should be acknowledged that all three behavioural tests require face matching, where face matching refers to the ability to judge whether two images of a face are from the same person, in addition to face perception. It is possible that autistic individuals are able to form an accurate perceptual representation of faces, but use sub-optimal decision criteria when deciding whether two facial images are from the same individual. If this is the case, it would be possible for face perception to be intact but poor performance to be observed on the OFMT and GFMT. Such a possibility remains to be investigated, but would be consistent with claims of a difficulty generalizing from exemplars in autism (Scherf et al., 2008).
